# Physiological and Pathological Responses of Grass Carp (*Ctenopharyngodon idella*) to Total Dissolved Gas Supersaturation During Exposure and Subsequent Recovery

**DOI:** 10.3390/ani16193039

**Published:** 2026-09-27

**Authors:** Xiaoqing Liu, Qiang Zhang, Xiaohan Luo, Fei Wang, Haoran Shi, Yao Yang, Yuanyi Zhang, Bing Yao

**Affiliations:** 1 School of Energy and Power Engineering, Xihua University, Chengdu 610039, China; 2 The Fishery Institute of the Sichuan Academy of Agricultural Sciences, Chengdu 611730, China; 3 Key Laboratory of Fluid and Power Machinery, Ministry of Education, Xihua University, Chengdu 610039, China

**Keywords:** total dissolved gas, *Ctenopharyngodon idella*, tissue damage, enzyme activity, recovery

## Abstract

The total dissolved gas (TDG) supersaturation induced by discharged floods severely threatens fish survival during the flood season. However, investigations into the physiological and pathological effects of TDG supersaturation on fish remain limited, and even less is known about the recovery from TDG-induced injuries at the physiological and pathological levels. The biochemical and histopathological responses of fish injured by TDG exposure, and whether injured fish can return to normal conditions, remain unclear. In this study, on the basis of a combination of in situ investigations and laboratory studies, we investigated the physiological and pathological responses to TDG supersaturation stress in grass carp, as well as their recovery after TDG exposure. The results revealed that 96 h of TDG exposure caused epithelial cell hyperplasia and necrosis in the gill tissues of grass carp, as well as mucosal epithelial cell necrosis in the intestine. However, the damaged gills failed to achieve restoration after a 336 h recovery period. No pathological changes occurred in the liver. Moreover, the present results revealed that under the above exposure conditions, TDG supersaturation significantly decreased catalase (CAT) activity and increased superoxide dismutase (SOD) activity, whereas no significant alterations in alanine aminotransferase (ALT) or aspartate aminotransferase (AST) activity were detected. The CAT and SOD activities of the species returned to normal levels after 24 h of recovery. Thus, our results suggest that the recovery of the impaired antioxidant system requires 24 h for species under relatively low TDG levels (≤120%), whereas histopathological recovery requires a longer period. These findings can be used to understand the potential effects of TDG exposure on fish and provide valuable information for formulating reservoir discharge plans.

## Simple Summary

Total dissolved gas (TDG) supersaturation caused by discharged floods is widely recognised as an environmental stress that threatens the health and survival of fish. Existing studies have focused mainly on the macroscopic effects of TDG on fish, while the etiology driving its effects on fish health and survival remains poorly understood. Furthermore, to date, few studies have systematically characterised the histopathological and biochemical recovery of fish injured by TDG exposure, and whether affected fish can return to normal physiological status after they experience TDG stress remains unclear. Thus, in this study, field investigations and laboratory experiments were combined to investigate the potential effects of TDG supersaturation on grass carp (*Ctenopharyngodon idella*) via biochemical and histopathological analyses, and the degree and duration of recovery in injured fish were quantified. The results revealed that TDG supersaturation (120%) induced tissue damage, triggered oxidative stress, and disturbed the antioxidant system, whereas these adverse effects were not observed at the 115% TDG level. The impaired antioxidant system could be restored after 24 h of recovery. However, the damaged gills failed to achieve restoration after a 336 h recovery period. To mitigate the adverse effects of TDG supersaturation on grass carp, a sufficient recovery duration (>336 h) and TDG level control (≤115%) should be considered in the formulation of reservoir discharge schemes. These findings advance the understanding of the physiological effects of TDG supersaturation on fish. Importantly, this study addresses significant gaps in the existing literature regarding the recovery of TDG-injured fish at the microscopic level.

## 1. Introduction

Hydroelectric engineering inevitably has adverse effects on the ecological environment while providing social and economic benefits such as flood control, power generation, irrigation, and water supply. During the flood season, flood discharge from high dams induces total dissolved gas (TDG) supersaturation in downstream river channels [1,2]. TDG supersaturation can cause fish to suffer from gas bubble disease (GBD) and even death. In 1968, more than 20,000 Chinook salmon (*Oncorhynchus tshawytscha*) died when discharged water released from John Day Dam led to TDG supersaturation [3]. In 2014, many fish deaths were observed in the Xiangjiaba hydropower station reservoir because of TDG supersaturation caused by flood discharge from Xiluodu Dam [4]. At present, TDG supersaturation is an eco-environmental problem of global concern.

In recent years, an increasing number of high dams, such as the Baihetan dam, Pubugou dam, and Xiangjiaba dam, have been constructed in the Yangtze River Basin. With the operation of numerous high dams, their impact on the ecological environment has attracted extensive attention. The impact of TDG supersaturation generated by high-dam discharge on fish is becoming an important ecological risk for the safe operation of these dams. Therefore, many studies have investigated the impact of TDG supersaturation on fish. Previous studies have indicated that many rare fish species, including *Schizothorax prenanti, Myxocyprinus asiaticus*, and *Procypris rabaudi*, exhibit low tolerance to TDG supersaturation [5,6]. The species (e.g., *Hypophthalmichthys molitrix* and *Hypophthalmichthys nobilis*) inhabit the upper layer of the water body and are prone to injury from TDG supersaturation due to GBD [7,8]. Some studies have reported that TDG supersaturation can lead to severe GBD, impair swimming performance, and disrupt migration and predation [9,10]. The growth and development of fish subjected to TDG stress are also inhibited [11,12]. However, previous studies have focused mainly on the macroscopic effects of TDG on fish, such as GBD, tolerance, swimming performance, and behaviour. These findings do not characterise the biochemical and histopathological responses of fish to TDG supersaturation stress.

In general, alterations in aquatic environments can impose substantial physiological stress on fish, potentially resulting in immune suppression, oxidative stress, metabolic disruption, and tissue damage [13,14,15]. Such physiological disturbances can be reflected by biochemical biomarkers. Superoxide dismutase (SOD) and catalase (CAT) are core antioxidant enzymes that are sensitive to the degree of oxidative stress and the functional status of the antioxidant defence system. In comparison, aspartate aminotransferase (AST) and alanine aminotransferase (ALT) are classic biomarkers for evaluating tissue injury and metabolic dysfunction. However, TDG supersaturation is considered environmental stress, and research on the histopathological and physiological changes in fish under these stress conditions is limited. The extent of tissue damage and the underlying biochemical changes that influence fish health and survival remain poorly understood. Furthermore, most studies have been conducted under controlled laboratory conditions with constant TDG levels, which cannot reflect the fluctuations in TDG saturation and complex hydrodynamic conditions in natural water bodies when floods occur [16]; however, this finding creates limitations in evaluating the ecological risk for the safe operation of high dams. At present, in situ studies investigating the effects of TDG supersaturation on fish are still lacking, especially in terms of physiological responses and pathological injuries.

Previous studies have characterised the phenotypic recovery of fish from TDG supersaturation exposure in terms of swimming performance and GBD development [9,17]. However, to date, few studies have investigated the recovery of injured fish subjected to TDG exposure at the histopathological and physiological levels. It remains unclear whether fish can return to normal physiological conditions after they experience TDG supersaturation stress. Clarifying the recovery pattern is not only critical for evaluating the long-term survival risk of fish after TDG exposure but also provides a scientific basis for formulating ecological operation schemes.

Grass carp, as one of the four major carp in China, is widely distributed in the Yangtze and Pearl River Basins. It frequently occurs in near-shore shallow habitats for foraging, rendering it vulnerable to TDG supersaturation during dam spill events. In addition, the grass carp serves as a well-established cyprinid model in aquatic ecotoxicology research. Beyond its high economic value, this species has substantial scientific value. In the present study, the main goals were (1) to investigate the potential effects of TDG supersaturation on grass carp via biochemical and histopathological analyses, combining in situ investigations and laboratory experiments, and (2) to determine whether injured fish can return to normal physiological status and to quantify the degree and duration of their recovery. The results of this study are expected to complement the physiological mechanism of TDG supersaturation toxicity to fish and provide scientific support for ecological risk assessment and fish conservation during high levels of dam flood discharge.

## 2. Materials and Methods

### 2.1. Experimental Fish

In this study, grass carp were obtained from local farms. The mean weight ± standard deviation (SD) was 47.6 ± 15.9 g, and the mean body length ± SD was 16.1 ± 1.8 cm.

### 2.2. In Situ Study

An in situ study of TDG exposure was conducted in a backwater zone on the left bank of the Dadu River from 10 to 14 September 2023. The study site lies 6.5 km downstream of the Gongzui Dam on the Dadu River (Figure 1), a tributary of the Yangtze River, in Ebian County, Sichuan Province. In this experiment, two cages (length × width × height: 1 m × 1 m × 1 m) were employed (Figure 1). These cages were secured with ropes to a boat moored at the shore and fixed by a floating bowl to keep them floating on the surface of the water. The draft depth of these cages was maintained within the range of 0–1 m. Before the experiment, the grass carp were reared in aerated river water to adapt to the new environment. The water temperature and dissolved oxygen (DO) concentration were approximately 20 ± 0.5 °C and 10 ± 0.3 mg/L, respectively. After acclimation, 40 healthy juvenile grass carp were divided into the above two cages and exposed to TDG-supersaturated water in the river for 96 h. In addition, 20 healthy grass carp were transferred to aerated river water (100% TDG) as the control group. During the test, the TDG level was monitored with a TDG pressure meter (OxyGuard Inc., Farum, Denmark) at the water surface. The DO level, temperature, and turbidity level were measured with a multiparameter sonde (6600, YSI, Inc., Ohio, USA). These parameters were monitored every 8 h. At 48 h and 96 h, six fish were randomly sampled from the TDG group and six from the control group for subsequent analyses.

### 2.3. Laboratory Study

Laboratory studies were performed at the Key Laboratory of Fluid and Power Machinery, Xihua University. They included a sublethal TDG exposure experiment and subsequent recovery experiments. In laboratory studies, TDG-supersaturated water could be maintained on the basis of the test system used in a study by Wang et al. [18]. The fish were placed in tanks (length × width × height: 70 cm × 40 cm × 40 cm) with a TDG-supersaturated water depth of 40 cm. Furthermore, the TDG level, temperature, and DO level were monitored with the instruments used in the in situ study. The temperature and DO level were as consistent as possible with those observed in the in situ study.

Sublethal TDG exposure experiment—In the experiments, two TDG levels (115% and 120%) were used as the TDG groups. Equilibrated water constituted the control group (100% TDG). Before the experiment, the grass carp were reared in aerated water to adapt to the new environment. After acclimation, 90 healthy test fish were randomly divided into the TDG group (*n* = 45 per group) for 96 h. Twelve test fish were placed into the control group (100% TDG). During the entire exposure period, at each time point (48 and 96 h), six fish were randomly selected from the TDG group and the control group.

Recovery experiment—After the above sublethal TDG exposure experiment, the remaining test fish in the 115% and 120% TDG groups were transferred into two separate tanks with equilibrated water for the recovery experiment. The recovery period was 14 d (336 h). At each time point (24, 48, 168, and 336 h), six fish were sampled from the above two recovery tanks at 115% and 120% TDG. No control group was maintained during the recovery phase. All recovery-phase data were compared with the data from the control group in the above sublethal experiments.

### 2.4. Sampling Procedure

For in situ investigations and laboratory studies, an anaesthetic (MS-222; Sigma, St. Louis, MO, USA) was used at 70 mg/L to anaesthetise selected fish for subsequent sampling. After the experimental fish were euthanised, dissection was performed, and intestinal, gill, and liver tissues were collected and fixed in 4% paraformaldehyde (Labgic Technology Co., Ltd., Beijing, China) for histopathological analysis. Blood samples were extracted from the caudal vein of the fish and subsequently centrifuged using a high-speed freezing centrifuge (Lichen-BX Instrument Technology Co., Ltd., Shanghai, China) at 3000 rpm (10 min) and 4 °C. The enzymatic activity of the supernatant was measured for biochemical analysis.

### 2.5. Biochemical Analysis and Histopathological Assay

The activities of aspartate aminotransferase (AST, kit catalogue No. C010), alanine aminotransferase (ALT, kit catalogue No. C009), superoxide dismutase (SOD, kit catalogue No. A001), and catalase (CAT, kit catalogue No. A007) were measured by using assay kits (Nanjing Jiancheng Bioengineering Institute, Nanjing, China). For histological analysis, liver, gill, and intestinal tissues were removed from the paraformaldehyde and trimmed to the appropriate sizes. The tissue samples were dehydrated in an ascending graded ethanol series, followed by clearing in xylene (dimethyl benzene) for 35 min prior to paraffin embedding. These treated tissues were transferred into molten paraffin for sufficient wax infiltration. After wax infiltration was complete, each tissue sample was sectioned into 5 μm-thick slices and flattened with glass slides. The dried slides were subsequently dewaxed and cleaned. After being stained with haematoxylin-eosin for 30 min, the slides were dehydrated, cleared, and mounted for histological examination.

Histopathological lesions were evaluated using a five-grade scoring system (0–4), in which 0 = normal, 1 = mild, 2 = moderate, 3 = severe, and 4 = extreme [19].

### 2.6. Data Analysis

Analysis of variance was used to compare the changes in the serum enzymatic activity. Differences among the different treatment groups were assessed via post hoc multiple comparison tests. Duncan’s test was employed when the assumption of homogeneity of variance was met, whereas Tamhane’s T2 test was used when this assumption was not satisfied. Comparisons between different TDG groups at the same time point were performed by using a *t* test. *p* < 0.05 indicated a significant difference. The data are presented as the means ± standard deviations.

## 3. Results

### 3.1. In Situ Experiment

In the in situ experiment, the average values of the TDG level, DO concentration, water temperature, and turbidity were 119.6 ± 1.1% (range: 117.6–121.0%), 10.3 ± 0.2 mg/L (range: 10.0–10.5 mg/L), 19.6 ± 0.6 °C (range: 18.7–20.4 °C), and 47.0 ± 5.8 NTU (range: 34.1–53.4 NTU), respectively (Table 1). During the experiment, no signs of mortality or gross disease were detected in the grass carp.

Pathological changes were observed in grass carp after 96 h of exposure to TDG-supersaturated water in the natural river (Figure 2). The gills exhibited epithelial cell hyperplasia, necrosis, and interlamellar cell proliferation, which resulted in lamellar fusion in the TDG group (Figure 2B). The intestine exhibited mucosal epithelial cell necrosis (Figure 2D). The liver showed no lesions (Figure S1).

The changes in enzyme activity in grass carp are shown in Figure 3. No significant differences in ALT or AST activity were detected between the control group and the TDG exposure groups at 48 and 96 h in the natural river (Figure 3). CAT activity significantly decreased at 48 and 96 h of exposure, whereas SOD activity significantly increased.

### 3.2. Laboratory Experiment

After 96 h of TDG exposure in the laboratory, no lesions were observed in the gill tissue of grass carp at 115% TDG (Figure 4A,B). At 120% TDG, gill lesions, including those with epithelial cell hyperplasia, inflammation, and interlamellar cell proliferation, were detected (Figure 4C). After 336 h of recovery, these damaged gills failed to achieve restoration (Figure 4D). In addition, no damage was observed in the intestinal or liver tissue during the laboratory experiments (Figure S2).

The changes in enzyme activity in grass carp in the laboratory are shown in Figure 5. No significant differences were observed in the activities of ALT and AST (Figure 5A,B). With respect to CAT activity, significant decreases were detected at 48 and 96 h in the 120% TDG group (Figure 5C), and significant differences were detected between the 115% and 120% TDG groups at 48 and 96 h. Grass carp experienced 24 h of recovery in equilibrium water, and the CAT activity in the 120% TDG group reached a level comparable to that in the control group. With respect to SOD activity (Figure 5D), significant increases were observed at 48 and 96 h in the 120% TDG group. Compared with that in the 115% TDG group at the above exposure times, the SOD activity significantly increased in the 120% TDG group. As shown in Figure 5D, the SOD activity in the 120% TDG group was comparable to that in the control group when the grass carp were allowed to recover for 24 h in equilibrium water.

### 3.3. Comparison of the Results of the In Situ Study and Laboratory Study

The histopathological and physiological changes in grass carp after TDG exposure are shown in Table 2 and Table 3. As shown in Table 2, epithelial cell hyperplasia and necrosis were observed in gill tissues in the in situ study, and mucosal epithelial cell necrosis was detected in intestinal tissues. In addition, in a laboratory study, TDG exposure (120% TDG) caused epithelial cell hyperplasia and inflammatory cells. In the above two studies, no lesions were detected in the liver tissue.

As shown in Table 3, ALT and AST activities did not significantly change, and similar results were obtained in the in situ study (119.6% TDG) and laboratory study (120% TDG). Similarly, for CAT and SOD activities, the results of the in situ study (119.6% TDG) were consistent with those of the laboratory study (120% TDG), and these enzyme activities significantly changed in the above two studies compared with those of the control group. Furthermore, there was no significant difference between the 115% TDG group and the control group (100% TDG).

## 4. Discussion

### 4.1. Tissue Damage

Previous studies have shown that gills are susceptible to environmental stressors caused by alterations in water quality [20,21]. For instance, benzo[α]pyrene exposure induced varying degrees of DNA strand breaks in the gill cells of Caspian whitefish [21]. Gill lamellar aneurysms and secondary epithelial hyperplasia were observed in the gills of goldfish (*Carassius auratus*) and yellow catfish (*Pelteobagrus fulvidraco*) under environmental exposure, and the severity of lesions increased with increasing exposure levels [22,23]. In our study, the in situ investigation revealed that 96 h of TDG exposure (average 119.6%) caused epithelial cell hyperplasia and necrosis in the gills of grass carp in a natural river during flood discharge. The results of laboratory experiments revealed that TDG supersaturation (120% TDG) led to similar pathological damage to the gills of the species at the same exposure time. Although grass carp may perform compensatory vertical diving to deeper waters to partially mitigate the adverse effects of TDG-supersaturated water [24], the consistent results from both field and laboratory studies confirm that TDG supersaturation can induce significant gill lesions in grass carp. In addition, our recovery experiment revealed that gill tissue damage persisted even after grass carp were transferred to TDG-equilibrated water (100% TDG) for a 336 h recovery period. These observations indicate that TDG-induced gill injury is not rapidly reversible and may have prolonged effects on fish respiratory physiology and health. Notably, however, the physiological recovery observed under laboratory conditions may not fully reflect fish recovery responses under variable hydrodynamics and fluctuating TDG levels in natural rivers. This limitation of our work should be considered when extrapolating our laboratory recovery results to field scenarios. Thus, further research is needed to elucidate the complete recovery process and verify whether damaged gill tissue can restore normal morphology and function, with additional field investigations encouraged.

In general, the liver, as a vital organ, plays a crucial role in the physiological response to environmental stressors in fish, and such stressors are widely documented to induce liver tissue damage in fish [25,26]. For instance, gas bubble disease triggered histopathological alterations in fish liver, including vacuolisation, necrosis, and cordal disarray [27]. As a widespread abiotic stressor associated with dam discharge, total dissolved gas (TDG) supersaturation can cause gas bubble formation and vascular blockage (gas embolism), as well as systemic oxidative stress and metabolic disorders in fish, which may further trigger pathological impairment of the liver. In the present study, no pathological lesions were detected in the liver tissues of grass carp following exposure to TDG supersaturation in both in situ surveys and laboratory experiments. Nevertheless, pathological research focusing on the adverse effects of TDG supersaturation on fish remains limited. Therefore, further systematic investigations are warranted to clarify whether TDG supersaturation can trigger hepatic damage in fish. It is reasonable to hypothesise that TDG supersaturation may induce hepatic lesions in fish species with weaker tolerance to TDG supersaturation. The lack of observable hepatic injury in the present study is likely attributed to the relatively low TDG levels used in our exposure treatments. Future studies could establish wider TDG level gradients and extend the exposure duration to explore the dose-effect relationship between TDG supersaturation and hepatic damage in fish.

The intestine is important for digestion, nutrient absorption, and mucosal immune defence in fish. Previous studies have confirmed that environmental stressors can disrupt intestinal redox homeostasis and induce oxidative damage to intestinal tissues [28,29], whereas the intestinal epithelium can synthesise endogenous antioxidants to counteract oxidative injury and maintain mucosal barrier integrity [20,21,22,23,24,25,26,27,28,29,30,31,32]. With respect to exposure to TDG supersaturation, gas bubbles formed in the intestinal lumen may compress the mucosal layer and damage epithelial cells, which may further trigger oxidative stress and pathological damage in the intestine. However, excess gas may escape through the mouth or anus, potentially limiting the persistence and severity of such damage. A prior study revealed abundant gas bubbles in the intestinal tract of *Pelteobagrus fulvidraco* after 48 h of exposure to 116% TDG supersaturation; however, whether this condition was accompanied by histopathological alterations in the intestine was unclear [23]. In our in situ investigation, mucosal epithelial necrosis was observed in the intestinal tissues of grass carp under TDG supersaturation stress, whereas no lesions were detected in the laboratory study. A possible explanation is that environmental cofactors present in the in situ experiment (e.g., turbidity, flow velocity, or suspended particles) amplified intestinal damage, as previous studies have demonstrated that suspended sediment and water velocity can cause histopathological alterations in the intestinal tissues of zebrafish [33] and coral trout [34]. Combined with existing evidence [23], these results suggest that different fish species exhibit distinct intestinal response patterns to TDG supersaturation: bubble formation is the prominent phenotype in *Pelteobagrus fulvidraco*, while epithelial cell necrosis is the primary pathological manifestation in grass carp. These interspecific differences may be attributed to variations in intestinal physiological structure and antioxidant defence capacity among fish species. From an ecological perspective, intestinal epithelial necrosis can impair nutrient absorption efficiency and mucosal immune function in fish, which may further reduce individual growth and disease resistance under long-term TDG supersaturation exposure. Our findings provide additional histopathological evidence of TDG-induced intestinal damage in fish.

### 4.2. Influence of TDG Supersaturation on Enzyme Activities

SOD and CAT are critical antioxidant enzymes that play key roles in the antioxidant defence system of fish [35]. They act synergistically to eliminate oxygen free radicals and prevent oxidative damage caused by pollutants, pathogens, or drastic environmental changes [36]. In the present study, SOD activity increased in the in situ investigation (average 119.6% TDG) and laboratory experiment (120% TDG), whereas CAT activity decreased after 96 h of TDG exposure. These findings indicate that TDG supersaturation stress induces oxidative stress in grass carp.

As the first line of antioxidant defence, SOD is rapidly induced to decompose superoxide anions into H_2_O_2_ and O_2_ [37]. The created H_2_O_2_ was subsequently decomposed into harmless substances (H_2_O and O_2_) by CAT [38]. In our study, the elevated SOD activity reflected an adaptive response to eliminate excess reactive oxygen species, whereas the decreased CAT activity suggested that the H_2_O_2_ scavenging capacity was overwhelmed under sustained stress. An imbalance between SOD and CAT leads to H_2_O_2_ accumulation and further oxidative damage to cellular components. Similar response patterns of SOD and CAT were reported in *A. dabryanus* after 48 h of TDG exposure (116% TDG) [39]. Our findings suggest that TDG supersaturation can disrupt the antioxidant defence system of grass carp.

Furthermore, for the first time, we investigated the recovery of injured fish subjected to TDG exposure at the microscopic level. The results of this study revealed that the CAT and SOD activities of grass carp exposed to TDG supersaturation (120% TDG) returned to normal levels after 24 h of recovery in equilibrated water (100% TDG), indicating that the antioxidant system may have been restored for test fish that were provided with a 24 h recovery time. Notably, a limitation of our recovery study is the absence of a time-matched control group during the recovery phase. All recovery-phase data were compared with the data from the control group in the sublethal experiment. Although the gradual convergence of enzyme activities towards the baseline suggests that TDG-triggered oxidative stress appears to be reversible, potential baseline fluctuations induced by fasting or prolonged captivity cannot be fully excluded. This factor should be considered when evaluating the recovery capacity of grass carp.

AST and ALT are two key aminotransferases involved in hepatic amino acid metabolism, and their elevated activities are recognised as sensitive biomarkers of liver damage and metabolic disturbance induced by environmental stress [40]. These aminotransferases are generally used as indicators to assess physiological responses in fish exposed to environmental stresses, such as pollutants or pathogens [25,41]. For instance, largemouth bass and rainbow trout subjected to environmental stress exhibit increased ALT and AST activities [13,15]. In our study, no significant alterations in ALT or AST activity were detected in grass carp under TDG supersaturation stress in the in situ investigation (average 119.6% TDG) or in laboratory experiments (120% TDG). These findings suggest that relatively low levels of TDG may not cause apparent liver damage or metabolic disturbances in grass carp, and this inference is further supported by our histological observations. However, divergent results have been reported in other fish species: A previous study showed that 48 h of exposure to 116% TDG supersaturation caused significant changes in ALT and AST activity in *Schizothorax davidi* [42]. This inconsistency may be attributed to differences in metabolic regulation patterns and TDG tolerance among fish species.

### 4.3. Limitations and Perspectives

In general, discharged floods can cause high TDG levels (120–143%), and the TDG level (≤120%) was relatively low in our study. To better understand the pathological and physiological effects of TDG exposure on fish, further studies at high TDG levels (>120%) are necessary in the future. In addition, compared with the grass carp used in our study, which lived in the middle and lower layers of water, the pelagic fish were more susceptible to damage from TDG supersaturation. More studies should be performed to determine the effects of TDG exposure on pelagic fish or other species with poor tolerance and weak ability to avoid TDG stress. At present, few studies have reported the recovery of injured fish subjected to TDG exposure at the microlevel, and in-depth studies are needed at the molecular level, particularly for endangered and weakly tolerant species. Although our results provide valuable information for understanding the effects of TDG exposure on fish, explanations of the underlying mechanisms are limited, and further studies should be carried out to investigate the underlying molecular mechanisms via proteomics, transcriptomics, and metabolomics analyses.

## 5. Conclusions

In this study, the results revealed that TDG supersaturation caused gill damage in grass carp after 96 h of TDG exposure (approximately 120%), whereas no pathological changes occurred in the liver. The activities of antioxidant enzymes (CAT and SOD) changed significantly at this saturation level, indicating that TDG stress induced oxidative stress in grass carp and may have disrupted the antioxidant system. These enzyme activities returned to normal levels within 24 h of recovery, suggesting that the activity of the antioxidant system may be restored. However, following a 336 h recovery period, the damaged gills failed to recover. This finding indicates that recovery of the antioxidant system does not guarantee full recovery of respiratory tissues, which require a longer repair period. No significant alterations in aminotransferase (ALT and AST) activity were observed in grass carp subjected to TDG exposure (≤120%), indicating that relatively low levels of TDG may not cause apparent liver damage or metabolic disturbances in grass carp. Furthermore, our results revealed that at the 115% TDG level, neither tissue damage nor alterations in enzyme activity were induced. Therefore, we suggest that TDG supersaturation exhibited few pathological or physiological effects on grass carp when the TDG level is ≤115%. To mitigate the adverse effects of TDG supersaturation on grass carp, a sufficient recovery duration (>336 h) and TDG level control (≤115%) should be considered in the formulation of reservoir discharge schemes.

## Figures and Tables

**Figure 1:**
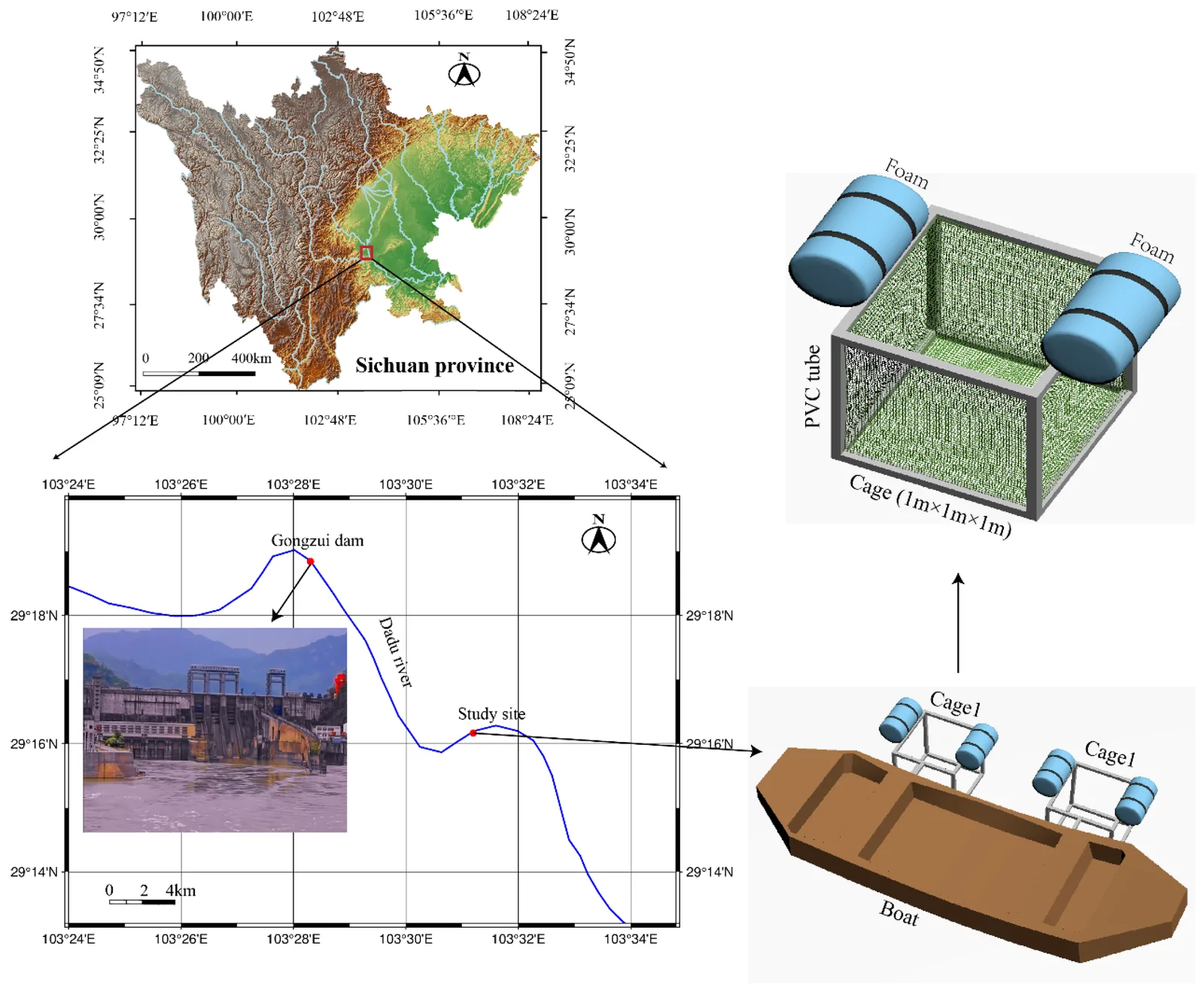
Map of the study site.

**Figure 2:**
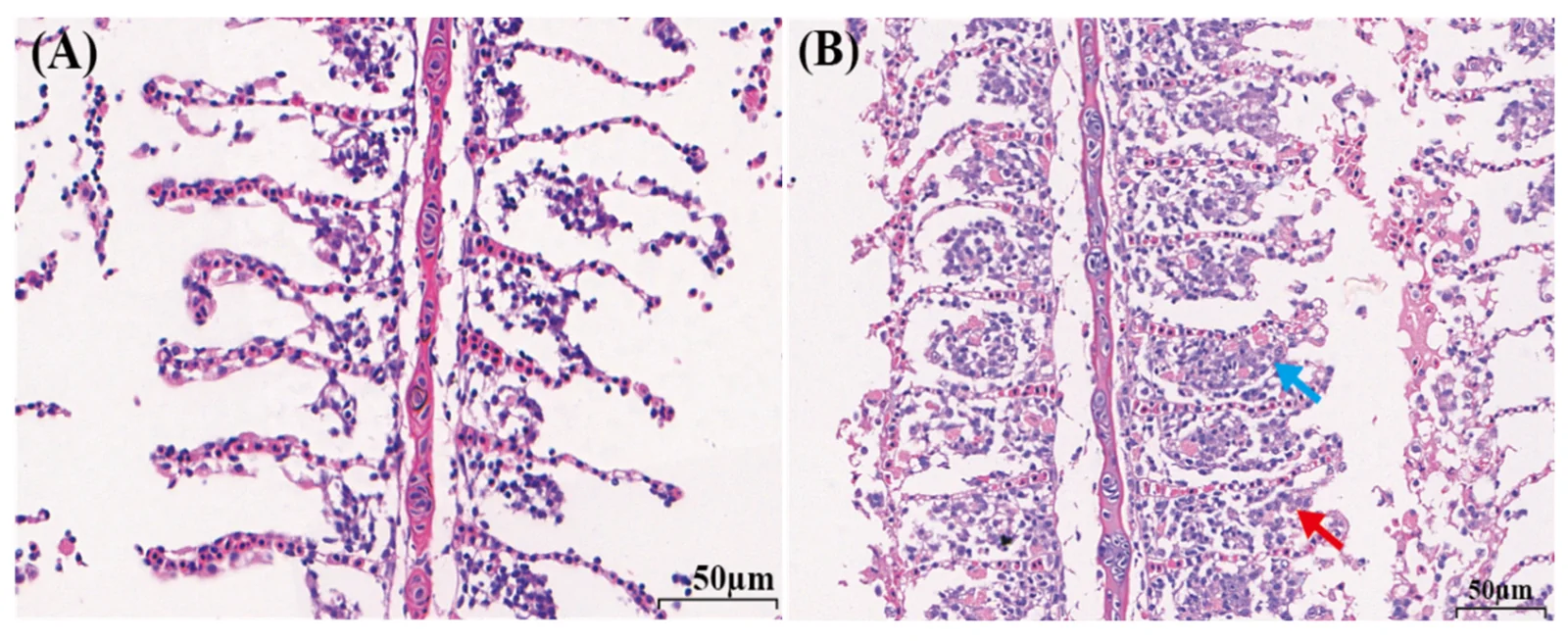

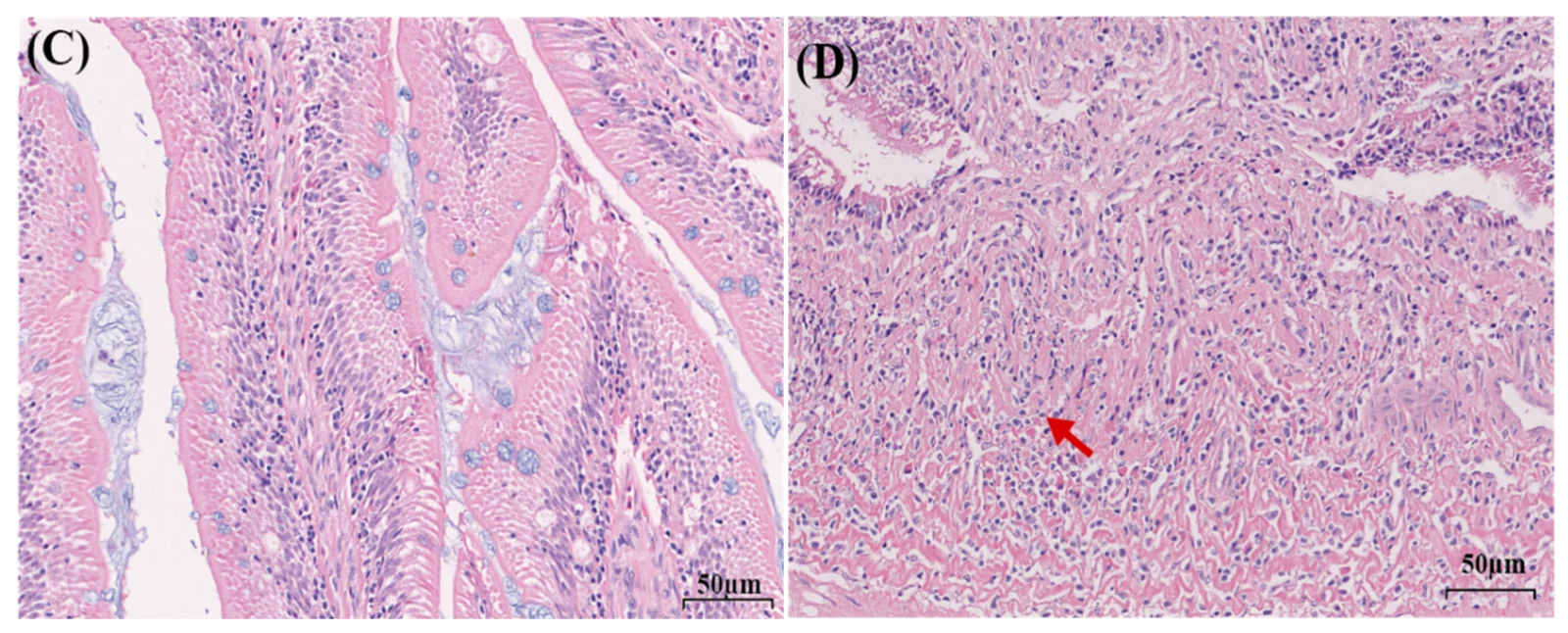
Histological alterations in tissues. (**A**) Gill tissue in the control group. (**B**) Gill tissue in the TDG group. (**C**) Intestinal tissue in the control group. (**D**) Intestinal tissue in the TDG group. The blue arrow represents epithelial cell hyperplasia; the red arrow represents epithelial cell necrosis ((**A**–**D**): 400× microscopic magnification).

**Figure 3:**
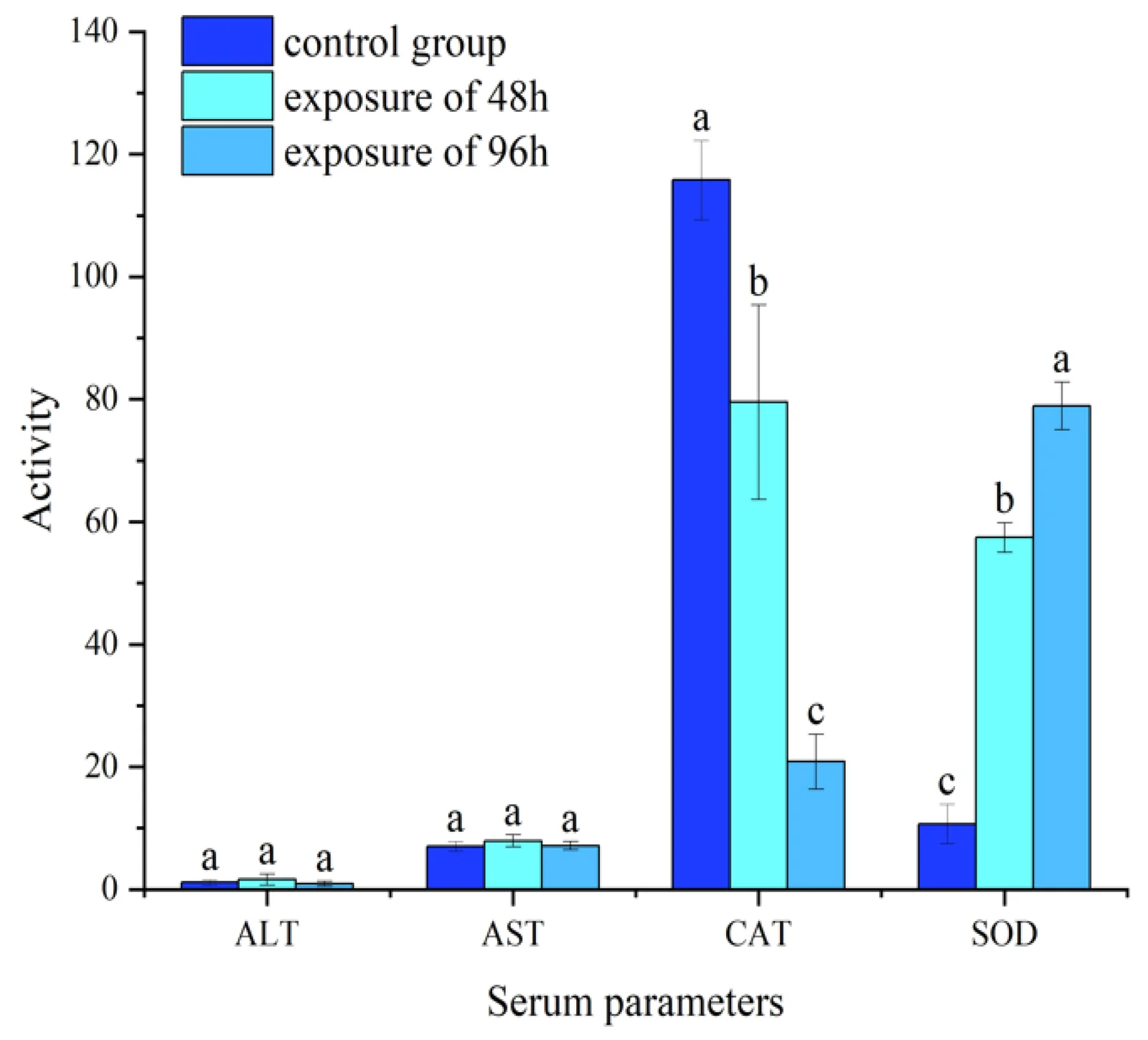
Activities of ALT, AST, CAT and SOD in grass carp after TDG exposure in the natural river. Different lowercase letters indicate significant differences between groups. The data are presented as the means ± standard deviations. Units: ALT and AST (U/L); CAT and SOD (U/mL).

**Figure 4:**
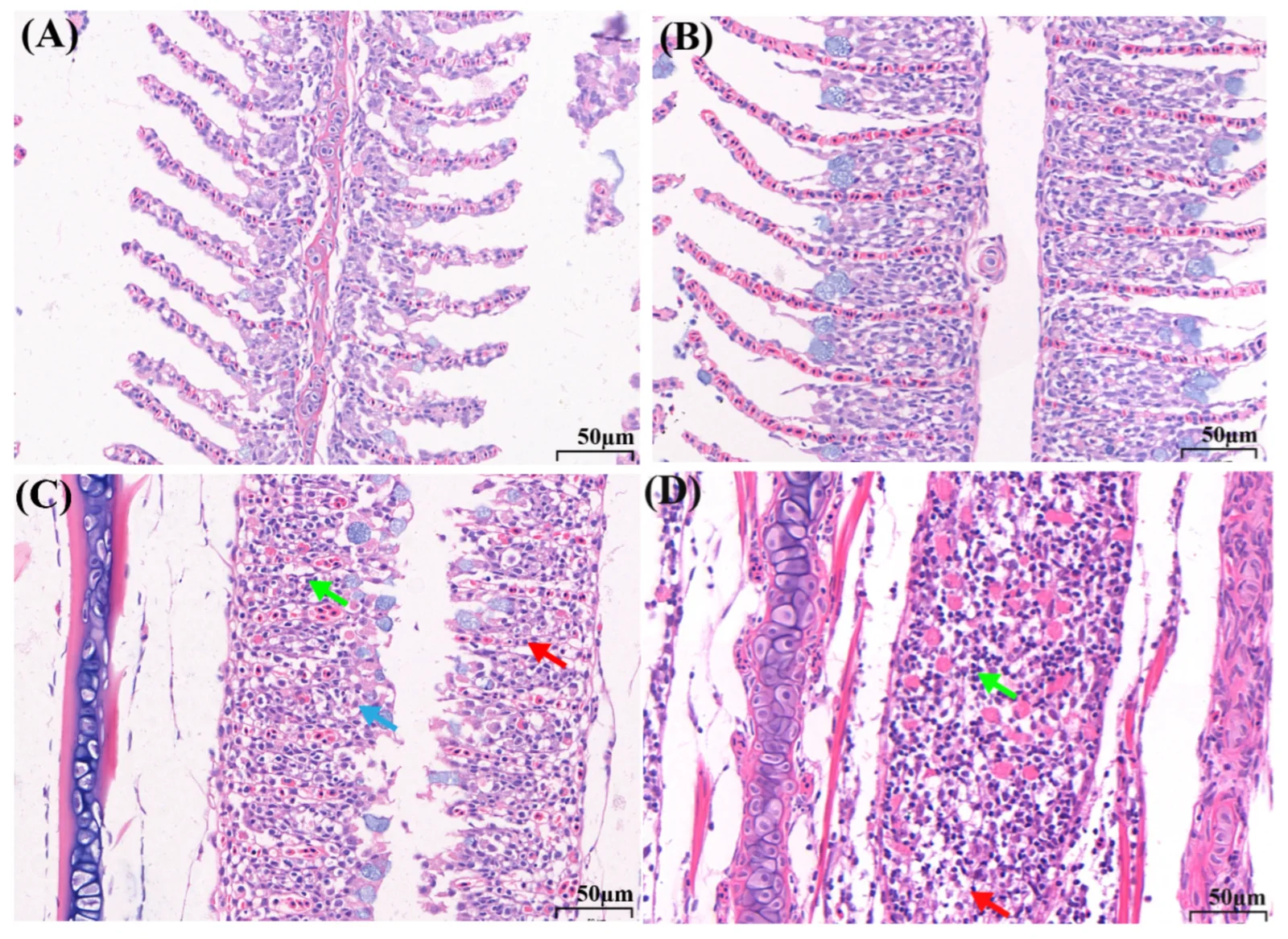
Histological alterations in tissues at different TDG levels after 96 h of exposure and subsequent recovery. (**A**) Gill tissue in the control group; (**B**) gill tissue in the 115% TDG group after 96 h of exposure; (**C**,**D**) gill tissue in the 120% TDG group after 96 h of exposure and after 336 h of recovery; the blue arrow represents epithelial cell hyperplasia; the green arrow represents inflammatory cells; and the red arrow represents epithelial cell necrosis. Magnification: 400×.

**Figure 5:**
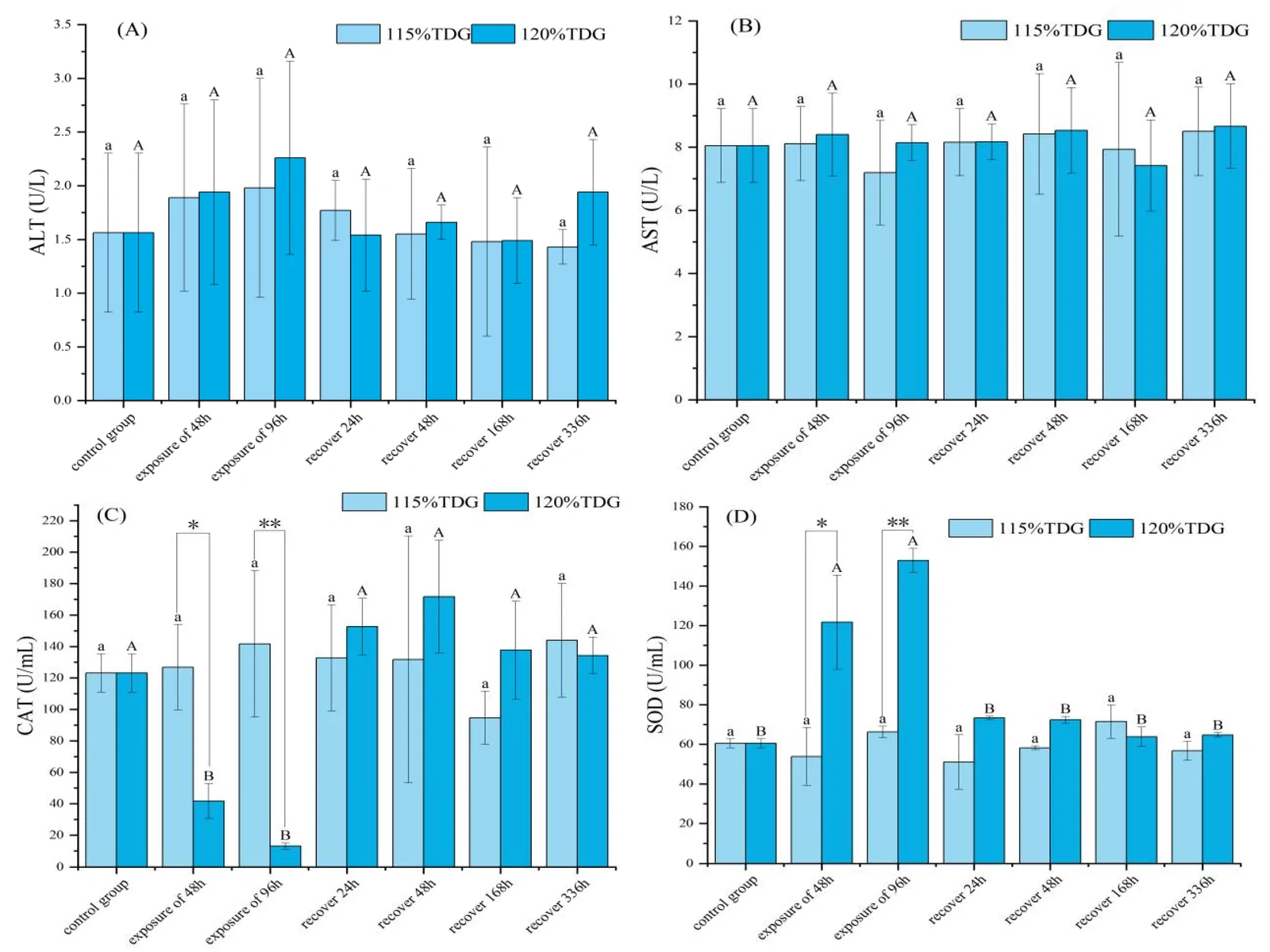
Serum biochemical parameters of grass carp in the exposure and recovery experiments. (**A**) ALT activity; (**B**) AST activity; (**C**) CAT activity; (**D**) SOD activity. Values that do not share a common letter differ significantly at *p* < 0.05 (lowercase letters for the 115% TDG group and uppercase letters for the 120% TDG group). The values of activity are displayed as the means ± standard deviations. Asterisks indicate significant differences between TDG levels at the same time point (* *p* < 0.05, ** *p* < 0.01).

**Table 1:** Temporal variations in TDG, DO, turbidity, and temperature at the study site.

Time(h)	Temperature (°C)	Turbidity (NTU)	DO (mg/L)	TDG (%)
0	19.8	42.0	10.4	120.2
8	20.3	48.5	10.4	119.8
16	19.3	51.2	10.3	119.0
24	19.0	49.2	10.2	120.6
32	19.8	53.0	10.0	117.6
40	18.7	53.4	10.1	118.3
48	18.7	34.1	10.4	120.5
56	19.4	50.8	10.2	119.5
64	19.9	47.3	10.5	121.0
72	20.4	39.1	10.3	118.8
80	19.6	51.5	10.5	120.7
88	20.1	48.0	10.2	117.9
96	20.2	43.4	10.4	120.5

**Table 2:** Histopathological changes in grass carp (*n* = 6) according to in situ and laboratory studies.

Tissues	TDG Level	Control Group	Exposure of 96 h
Gill	119.6% (in situ study)	No lesions	epithelial cell hyperplasia and necrosis ^2^
	115% (laboratory study)	No lesions	No lesions
	120% (laboratory study)	No lesions	epithelial cell hyperplasia and necrosis, inflammatory cells ^2^
Intestine	119.6% (in situ study)	No lesions	mucosal epithelial cell necrosis ^3^
	115% (laboratory study)	No lesions	No lesions
	120% (laboratory study)	No lesions	No lesions
Liver	119.6% (in situ study)	No lesions	No lesions
	115% (laboratory study)	No lesions	No lesions
	120% (laboratory study)	No lesions	No lesions

Note: 2 = mild lesions; 3 = moderate lesions.

**Table 3:** Physiological changes in grass carp in in situ and laboratory studies.

Enzymes	TDG Level	Control Group	Exposure of 48 h	Exposure of 96 h
ALT(U/L)	119.6% (in situ study)	1.13 ± 0.46	1.62 ± 0.91	0.93 ± 0.43
	115% (laboratory study)	1.56 ± 0.74	1.89 ± 0.87	1.98 ± 1.02
	120% (laboratory study)	1.56 ± 0.74	1.94 ± 0.86	2.26 ± 0.90
AST(U/L)	119.6% (in situ study)	1.36 ± 0.79	1.70 ± 0.98	1.13 ± 0.65
	115% (laboratory study)	8.05 ± 1.17	8.11 ± 1.17	7.19 ± 1.65
	120% (laboratory study)	8.05 ± 1.17	8.40 ± 1.31	8.14 ± 0.57
CAT(U/mL)	119.6% (in situ study)	115.89 ± 6.45	79.53 ± 15.86 *	20.85 ± 4.49 **
	115% (laboratory study)	123.15 ± 12.20	126.78 ± 27.18	141.66 ± 46.62
	120% (laboratory study)	123.15 ± 12.20	41.72 ± 11.03 *	13.09 ± 1.93 *
SOD(U/mL)	119.6% (in situ study)	10.76 ± 3.21	57.46 ± 2.42 **	78.91 ± 8.63 **
	115% (laboratory study)	60.51 ± 2.28	53.88 ± 14.65	66.27 ± 2.98
	120% (laboratory study)	60.51 ± 2.28	121.69 ± 23.75 *	152.92 ± 6.05 **

The values represent the means ± SDs. Asterisks indicate significance (* *p* < 0.05; ** *p* < 0.01).

## Data Availability

The datasets generated and analyzed during the current study are available upon request from the corresponding author.

## Supplementary Materials

The following supporting information can be downloaded at: https://www.mdpi.com/article/10.3390/ani16193039/s1, Figure S1: Histological alterations in the liver tissue in a natural river; Figure S2: Histological alterations in the intestinal and liver tissue in the sublethal TDG exposure experiment.
